# Dynamic and diverse amphibian assemblages: Can we differentiate natural processes from human induced changes?

**DOI:** 10.1371/journal.pone.0214316

**Published:** 2019-03-26

**Authors:** Nathália G. S. Lima, Ubirajara Oliveira, Rafael C. C. Souza, Paula C. Eterovick

**Affiliations:** 1 Programa de Pós-Graduação em Ecologia, Instituto de Ciências Biológicas, Universidade Federal de Minas Gerais, Belo Horizonte, Minas Gerais, Brasil; 2 Centro de Sensoriamento Remoto, Instituto de Geociências, Universidade Federal de Minas Gerais, Belo Horizonte, Minas Gerais, Brasil; 3 Programa de Pós-Graduação em Biologia de Vertebrados, Pontifícia Universidade Católica de Minas Gerais, Belo Horizonte, Minas Gerais, Brasil; Wildlife Conservation Society Canada, CANADA

## Abstract

Amphibians are sensitive to anthropogenic habitat alterations but also respond to natural drivers of assemblage composition at many levels. Additionally, they are usually hard to detect in field inventories. We used a multiscale approach, from microhabitat to the landscape levels, to try to understand the effects of natural changes, and try to distinguish them from the effects of landscape level anthropogenic changes, on dynamic and diverse anuran assemblages, taking imperfect detection into account. We conducted thorough field inventories in 16 streams at Serra do Cipó, in the southern portion of Espinhaço Mountain Range, southeastern Brazil, during two time periods separated by 16 years. We compared species richness and diversity between periods, sampling both tadpoles and adult frogs. We quantified tadpole microhabitat availability, alterations in immediate riparian vegetation, and changes in classes of land cover within buffers around streams (adult habitats) to test for their effects on species composition. We also tested for effects of human occupancy around streams on nestedness and turnover components of species diversity. Microhabitats and riparian vegetation explained some of the changes in species composition (or detection) between time periods. Nestedness seemed to be influenced by the stability of the landscape. Detectabilities were too low to support robust occupancy estimates for most species. Natural changes in local habitats occupied by anurans in montane meadows are likely to influence species distribution. Some species with robust estimates experienced change in their occupancy over the studied 16-year interval, although no anthropogenic causes could be directly associated with such changes. The low detectability of most species, even with thorough sampling effort, makes it very hard to detect amphibian declines and possibly tell them apart from natural population fluctuations. New techniques are needed that improve species detectability in such diverse tropical habitats.

## Introduction

At any given site, species assembly is driven by historical/evolutionary, environmental, biotic, and stochastic factors [1]. Anthropogenic impacts have been acting as an additional driver of species assembly by promoting local extinctions or species movements away from or to modified areas [2–3]. However, such impacts will act simultaneously with non-anthropogenic species assembly drivers with potentially synergetic effects. Thus, it is a challenge to isolate purely anthropogenic effects and evaluate their impact on biodiversity.

Amphibians have been recognized as the most threatened vertebrate group in the world, with hundreds of species facing imminent extinction [4–5]. Most threats suffered by amphibians have been associated to anthropogenic environmental changes [6] such as habitat loss, fragmentation, and degradation [4,7] as well as climate change [8]. Many studies have corroborated the effects of urbanization as being detrimental to amphibians [9–12], and even leading to population declines [13]. Amphibian species from lotic habitats have been the most affected in some regions such as Central America [6] and Brazil [14]. Amphibians are representatives of the current extinction crisis [4, 15–16], and can be good model organisms to study the effects of human impacts on biodiversity.

Several studies on populations or communities still use raw field-count data to infer parameters such as population size, presence/absence, or species richness [17–18]. The assumption of perfect and constant species detection probabilities is a flaw of these studies and subjects them to criticism [19,20]. Thus, robust statistical methods that account for variation in individual detection probabilities are an important tendency in species distribution studies [21–22], but have been applied to just a few groups of species from diverse tropical anuran assemblages (e.g., [23–24]).

Several studies that attempted to explain variation in amphibian assemblage spatial distribution (e.g., [9,11, 25–27]) did not encompass temporal scales adequate for species richness and composition to change considerably at either local or regional spatial scales. Besides, amphibians are known to vary greatly in detection probability, making solid community studies hard to accomplish [28]. This problem is likely to assume greater importance in tropical regions that shelter many rare species.

At broad spatial scales, it is also important to evaluate beta diversity and its components represented by nestedness (dissimilarity between sites caused by species loss) and turnover (dissimilarity caused by species replacement) [29–30]. In a landscape where turnover predominates all sites should be considered as potential candidates for conservation, whereas in landscapes where nestedness predominates sites with the greatest species richness should be given priority [30]. Habitat fragmentation is likely to increase beta diversity [31]. Impoverishment of small patches of habitat was found to lead to local species extinctions and increased nestedness for lizards and birds [32].

We studied stream anurans at Serra do Cipó, in the southern portion of the Espinhaço Mountain Range in Brazil, during two time periods separated by 16 years, when the area was characterized by increased human occupancy. We considered this scenario to represent an important challenge for the assessment of the effects of human interference on species assemblages at the landscape level. It is important because (1) the Espinhaço shelters several endemic anuran species [33], (2) highland stream frogs have shown a greater tendency to decline in many regions [5–6], and (3) we were able to accomplish very detailed and thorough field inventories separated by a 16-year interval that encompassed both natural and anthropogenic changes in the landscape. It is a challenge because the area shelters high species diversity with many species being rare (i.e., difficult to detect), and there is considerable spatial/temporal variation driven by natural attributes of streams and their riparian habitats [34] that could interact with human impacts to determine local species composition. It is important to stress that, although increased human occupancy can be noticed at the landscape level, little or no human alteration has been documented in the immediate riparian habitat or the studied streams themselves. Thus, the effects of human impacts on frog assemblages, if any, would be acting at the landscape level (e.g., hampering frog migration) [35] and ultimately affect local stream assemblages and beta diversity patterns. Our approach is thus important for understanding whether impacts detectable at a larger spatial scale can result in eventual population extinctions in local habitats that are apparently pristine.

**Fig 1:**
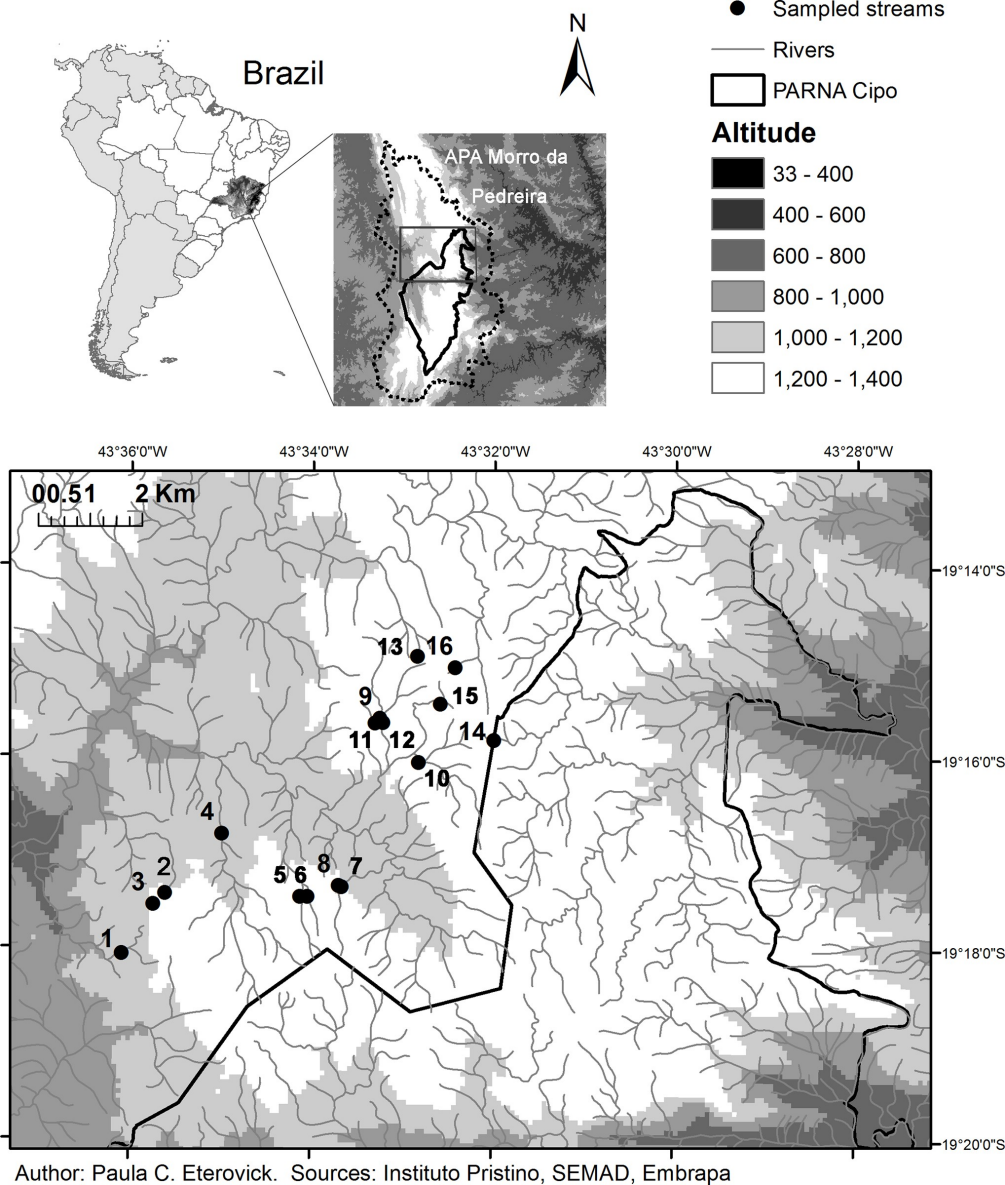
Study site. Location of the studied streams at APA Morro da Pedreira (dashed line) and PARNA Serra do Cipó in Minas Gerais State, Brazil.

We tested whether 16 years of increasing human occupancy in the landscape affected anuran species composition, richness, and diversity. We evaluated the effects of spatial and temporal natural changes in drivers of species assemblage composition at three spatial scales: (1) stream structure represented by tadpole microhabitat availability, (2) structure of the immediate riparian habitat represented by presence/absence of three vegetation strata, and (3) structure of the landscape surrounding streams. At the landscape level it was possible to quantify human occupancy and try to disentangle its effects on anuran species assemblages from those of natural causes (e.g., natural land cover changes, species local extinction and colonization dynamics). In the region, population structure data is available only for *Bokermannohyla saxicola* [36] and show that populations start to exhibit genetic differentiation at a distance of 2.5 km. Thus, the scale of our landscape approach (see Fig 1) is spatially adequate to encompass β-diversity variation as well as extinction/colonization processes.

We estimated detectability for all species in both sampling periods to evaluate whether changes in species composition/diversity could be due to incomplete sampling caused by imperfect detectability or whether it could be real. If real, such variation could be of trivial significance, being within the range of normal variation, or they could be outside the norm due to significant impact by humans. In this scenario, we expected increased anthropogenic land cover to impoverish overall habitat quality and increase nestedness. On the other hand, high turnover could reflect either species replacement resulting from natural species turnover or replacement of specialist/vulnerable by generalist/resistant species at impacted areas. Our approach differed from previous studies because we encompassed a range of temporal and spatial scales and also considered the influence of species detectability during interpretation of results.

## Materials and methods

### Study site

We compared local anuran assemblages at 16 streams between two different periods separated by 16 years in the highlands of the Serra do Cipó, in the southern portion of the Espinhaço Mountain Range, southeastern Brazil (Fig 1). The studied area encompassed the Serra do Cipó National Park (Parque Nacional—PARNA) and the surrounding protected area (Área de Proteção Ambiental–APA) Morro da Pedreira. According to the institution that regulates environmental protection in Brazil (Conselho Nacional do Meio Ambiente–CONAMA), PARNAs are fully protected from human activities except for tourism and research, whereas APAs are subject to some human activities regulated by local authorities. At the APA Morro da Pedreira in particular, roads have been paved and many houses and small hotels were built during our study period. Although activities such as fires, unauthorized vegetation removal/introduction and rock extraction are forbidden, they happen occasionally.

The PARNA Serra do Cipó and the APA Morro da Pedreira harbor the watersheds of the Doce and São Francisco river basins [37]. Their highlands (above 900 m above sea level) are covered mainly by montane meadows (Campos Rupestres), characterized by xeromorphic herbs and shrubs adapted to quartzitic soils with low capacity to retain water. Grasses dominate humid areas with deeper soil, and riparian forests can be found along the margins of some streams [38–39]. Campos Rupestres are considered an old climatically-buffered infertile landscape (OCBIL), which explains its megadiverse specialized vegetation [40]. OCBILs are, by definition, a cradle of continuing diversification of endemic lineages [40]. The climate is seasonal, with a dry season from April to September and a rainy season from October to March. Most rainfall (750–1600 mm a year) is usually concentrated within 3–4 months of the rainy season [39].

The sampled streams were chosen to represent a variety of riparian habitats and stream sizes present at the study site. The stream sections to be sampled were chosen in a way that the water from one sampled section would not reach another. The sections sampled during this study are at altitudes of 1059–1360 m above sea level and can be also considered representative of the microhabitats available to anurans at the streams of the study site, which vary on a smaller scale as a mosaic. Their bottoms are a mosaic of rocks, sand, and pebbles, with little aquatic vegetation at some places. Streambeds are irregular with many curves and exposed rocks, thus backwaters and rapids occur as a mosaic over the entire extensions of the sampled sections. The mosaic of microhabitats on stream bed can change with time due to natural movements of substrates, vegetation colonization and associated alterations in water movement. Predatory fish are absent, and the main tadpole predators are water bugs and Odonata naiads that are common in the streams [34]. Streams at the study site are oligotrophic, with 77–137% dissolved oxygen concentration, 5.5–6.4 μS/cm conductivity, total alkalinity around 0.03 mEq/L and low nutrient concentrations [41].

### Sampling of species

Previous inventories recorded 43 anuran species in our study area [42]. Of these, 27 breed in streams and have been recorded in at least one of the 16 study streams by one of us from 1998 to 2000 [34], representing our first sampling period. We re-sampled these same streams (at the exact same sections) with the same methods 16 years later to represent the second sampling period (2015–2016). We obtained permits from Sisbio/ICMBio (45318–1, 45318–2) and the Animal Ethics Comittee of PUC Minas (CEUA, 013/2015).

Field work consisted in 3 or 4-day sampling trips distributed throughout a year and a half in the first sampling period and one year in the second. These trips were distributed monthly during the rainy season (October-March) and every two months during the dry season (April-September), because most local species breed during the rainy season at the region [42]. In order to standardize sample effort for comparisons between sampling periods, we considered nine sampling trips (totalling 34 sampling days) per stream conducted during one entire year for each time period (referred to as 1998–1999 and 2015–2016 from here on).

Each sampling trip included one search during the day and another at night for each stream. These searches focused on all life stages (from eggs to adults) and were carefully and thoroughly conducted by two or three people along a 150 m section of each stream. We conducted visual and auditory searches encompassing the entire width of the streambed plus the riparian vegetation up to about two meters from each margin. We have experience in recognizing the calls of species that occur at the site, and we searched for calling males once we heard them. We also searched the entire section of the streams for silent individuals. Searches lasted at least two hours per stream and only ended when we considered that all possible microhabitats had been searched for specimens. The water of the streams is clear and we have experience in finding tadpoles visually and collecting them with dipnets, which we consider the most efficient method at the site. We checked by hand and with dipnets the few microhabitats that are harder to search visually due to greater depth, leaf litter or vegetation. In these circumstances, dislodged tadpoles can be easily seen and captured with dipnets.

We identified species in the field when we had no doubt of species identity. When this was not possible, we collected individuals for identification in the laboratory, which was the case for most tadpoles. One of the authors (PCE) has been working at the site for a long time and has previous experience with tadpole identification using a reference collection of Serra do Cipó tadpoles deposited at the Museum of Natural History of the Universidade Estadual de Campinas (ZUEC) by Dr. Ivan Sazima. We also used a key for tadpoles of the southern Espinhaço region [43]. Identification of adults was based on the previous experience of the authors and the literature [42]. Adult individuals that were collected for identification were euthanized in 10% lidocaine, fixed in formalin 10% and preserved in ethanol 70%. Tadpoles were euthanized in 10% lidocaine gel added to a small amount of water and preserved in a 50:50% mixture of ethanol 70% and formalin 10%. All collected specimens were deposited in the Amphibian Collection of the Museu de Ciências Naturais of the Pontifícia Universidade Católica de Minas Gerais (MCN-AM) and are presented as supplementary material (S1 Table).

### Sampling of habitat structure descriptors

We conducted additional visits to each studied stream to quantify microhabitat availability for tadpoles and adult frogs (based on occurrence of herbs, shrubs and trees in riparian habitats) following [44] and [34], respectively. We used 24 pre-defined microhabitat categories for tadpoles based on [44] for quantifying microhabitat availability (S2 Table).

In order to describe habitat structure of the landscape surrounding streams, we determined and quantified types of land cover within a buffer of 500 m around our sampled sections. Buffer size was based on mean core habitats reported for amphibians, which vary between 290 m [45] and 500 m [9] from stream margins. Buffers were aimed to represent an area of direct influence on anurans living in the stream and do not include the entire area covered by migrating individuals. For this purpose, we used Landsat 8 ETM (Enhanced Tematic Mapper) images (30 m resolution) obtained during the dry seasons of 1998 (11th August) and 2015 (12th June) from orbit 218 and point 73, with clouds covering from 0 to 40% of each image. The images were made available by the Instituto Nacional de Pesquisas Espaciais (INPE).

For image classification we established three real classes of land cover defined as: (1) anthropogenic (with intensive human use such as residences, pastures, silviculture, dirt and paved roads), (2) native forests, and (3) montane meadows. Types of land cover can change naturally according to patterns of plant growth and colonization. We used anthropogenic areas as a surrogate of human impact in the landscape because human impacts in the region (see Study Site) have not been directly identified and quantified regarding their potential negative effects on amphibians. We also used two additional classes for classification of areas that could not be analyzed, which corresponded to (4) clouds, and (5) shaded areas (areas shaded by clouds or relief, hiding spectral information). We conducted an Iso Cluster non-supervised classification of Landsat images in ArcGIS [46]. We then used the five classes in a Maximum Likelihood supervised classification of the 2015 image. We recorded coordinates of 50 random points classified in each real land cover category (anthropogenic, native forests, or montane meadows) and checked these points with Google Earth images. We used these data to build a confusion matrix that allowed us to calculate the Kappa index. The value of Kappa expresses the extent of agreement between reference and classification data [47], and the value obtained (0.65) indicated a good concordance (see [47]). We then used the same classification in the 1998 image. We could not use 1% of the images from 2015 due to the presence of clouds (no clouds covered the classified areas in the 1998 images). We could not use 0.29% of the images from 1998 and 7.9% of the images from 2015 due to shade that hampered land cover classification.

In order to posteriorly assess unbiased effects of land cover classes on amphibian assemblages, we tested for spatial autocorrelation of real land cover classes using Moran’s I coefficient [48]. Spatial autocorrelation could compromise the independency of the samples and bias analyses [49] possibly leading to false positives [50]. We conducted these analyses in the software SAM (Spatial Analysis in Macroecology) [51].

### Species richness, composition, and diversity

We first tested for a reduction in species richness between time periods that could have been caused by increased human impacts, as observed by [14] for a previous time interval at the same site. We estimated species richness based on species presence/absence data for each period using Jackknife I and Chao I indices in the software EstimateS 8.2 [52]. Our samples, standardized to 34 sampling days, yielded very different numbers of individual records (almost twice as many individuals recorded in 2015–2016 compared to 1998–1999). Thus, in order to evaluate the effect of different sampling success (which could be due to weather conditions or chance, for instance), we also compared species richness between sampling periods based on number of individuals (adult frogs and tadpoles, both together and separately) recorded in all streams using all the available data in rarefaction curves (based on 1000 simulations) in the software EcoSim [53]. Inventories including both adult and larval stages are more efficient for anurans, especially during a short time span [54]. However, numbers of individuals can vary among life stages. Thus, we evaluated both the contribution of larval and adult life stages to species inventories and also the effect of abundances, conducting all rarefaction curves with both abundance and presence/absence data.

Next, we tested for significant changes in species composition between time periods, regardless of alterations in species richness. We ordinated species records obtained in standardized inventories per stream and per period in a Non-Metric Multidimensional Scaling (NMDS) using the metaMDS function in the MASS package [55] of R [56]. We used the same data to test for spatial and temporal changes in anuran species composition with a PERMANOVA in the Vegan package [57] using streams and time periods as explanatory variables, respectively. We then tested our habitat descriptors [(1) number of records of each tadpole microhabitat type (obtained during microhabitat availability quantification), (2) number or records of each vegetation stratum at the immediate riparian habitat, and (3) percent cover of natural forests, montane meadows, and anthropogenic areas within 500 m buffers] as explanatory variables for changes in species composition. To evaluate changes in habitat structure, we used the same habitat descriptors in NMDSs and tested streams and time periods as explanatory variables with PERMANOVA.

Finally, we evaluated species β-diversity and the relative importance of turnover and nestedness to interpret the possible influence of anthropogenic impacts on changes in species diversity over space and time. We used a matrix of presence/absence of species in standardized inventories to calculate dissimilarity partitioning in the packages Vegan [57] and betapart [58] in R. In order to build this matrix, we considered whether we detected or not each species at each stream, at each sampling trip. The output includes three distance matrices: (1) the Sørensen index (β*sor*), which measures general variation in species composition; (2) a second index (β*sim*), which represents the fraction of species turnover, measured as Simpson dissimilarity; and (3) a third index (*βsne*), which represents the fraction of nestedness as the difference between β*sor* and β*sim*.

We made a map of land cover categories for each time period and overlapped the two maps to obtain classes of land cover change, represented by areas that (1) were natural (montane meadows or forests) in 1998 and remained natural in 2015 (NN), (2) were anthropogenic in 1998 and recovered to be natural in 2015 (AN), (3) were natural in 1998 and became anthropogenic in 2015 (NA), and (4) were anthropogenic in 1998 and remained this way in 2015 (AA). We tested whether these classes of land cover change correlated with changes in species diversity (β*sim*, β*sne*, and β*sor*) in streams between time periods using multiple regressions in R. We expected nestedness (β*sne*) to be positively related to increased human occupancy in the landscape (NA).

### Detectability and occupancy estimates

We used records of species presence/absence (considering all life stages) obtained during sampling trips to each stream to estimate both detectability and occupancy based on likelihood for each time period (according to [59]). For these analyses, we only included data from rainy seasons, when anurans are most active and likely to be detected, in order to avoid seasonal variation in detectability within each sampling period (see [60]). Adults registered and used for these analyses were mostly active calling males. We believe that all species included in occupancy estimates were actually using the streams to live/breed. We also found tadpoles for most species included in this analysis, if not within the rainy season, in the remaining months of the sampling period (in which case the tadpoles were not included in this analysis but were useful to prove that the species bred in the stream). This method assumes that (1) false negatives are possible (the species is present but not detected) but not false positives (the species is recorded when absent), (2) species detection in one stream is not influenced by its detection in any other stream, and (3) species distribution does not change during the sampling period evaluated (there are no extinctions or colonizations). We applied individual models of occupancy assuming constant occupancy *psi*(.) and detectability *p*(.), and disregarded effects of covariates, in the software PRESENCE version 11.3 (available at http://www.mbr-pwrc.usgs.gov/software/presence.html). We estimated the number of visits (N) necessary to record species presence at a stream based on detectability rates for each sampling period within the 95% confidence interval as N = log_10_ (1-a/100)/ log_10_ (1-p), where a = confidence interval (%) and p = detection probability [61].

## Results

### Species richness, composition, and diversity

**Fig 2:**
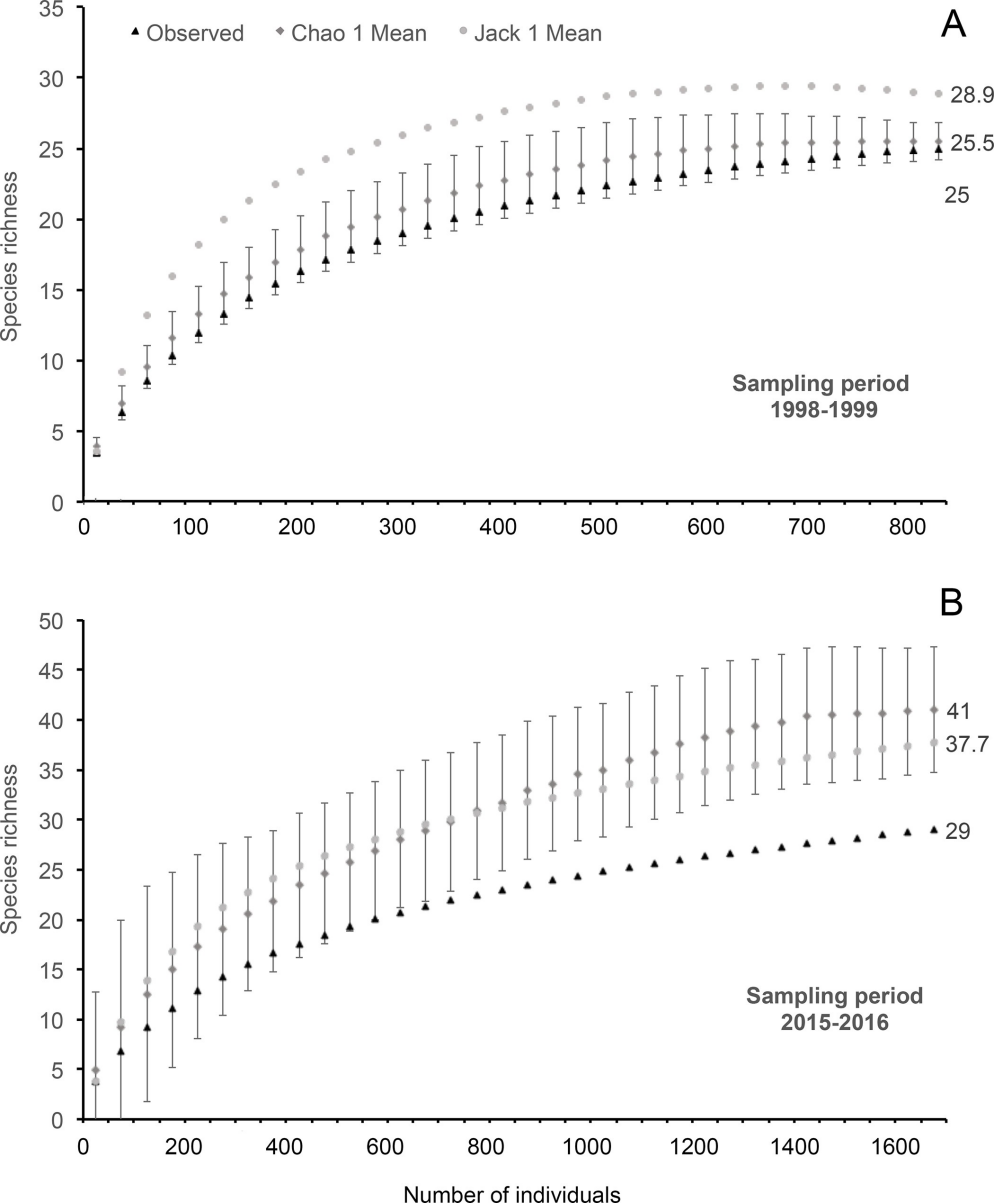
Rarefaction curves of cumulative anuran species richness based on Jackknife I and Chao I estimates for two time intervals at Serra do Cipó, southeastern Brazil. Analyses were conducted with data corresponding to 34 sampling days distributed throughout one entire year (see Materials and methods for details). Our complete sampling effort from 1998 to 2000 summed 1968 person hours distributed among 41 sampling days when 1059 individuals (735 tadpoles and 324 adult frogs) of 27 species were recorded. In 2015–2016, sampling effort summed 1632 person hours distributed among 34 sampling days when 1562 individuals (1276 tadpoles and 287 adult frogs) of 29 species were recorded.

The 1998–2000 inventory represented 70.7 and 76.8% of the estimated species richness while the 2015–2016 inventory represented 86.5 and 98%, according to Jackknife I and Chao I indices, respectively (Fig 2). Estimated species richness was higher in 2015–2016, however, the number of individuals recorded during this period was about twice that recorded in 1998–1999, despite equivalent sampling effort (34 sampling days distributed throughout an entire year) used in the analyses. Estimates generated by Chao I and Jackknife I indices were much closer to each other at comparable numbers of individuals (Fig 2). Rarefaction curves also showed species richness to be equivalent in both sampling periods (1998–1999 and 2015–2016) at a standardized sample size of 1059 individuals (estimated richness of 27 and 26 species respectively). Considering just tadpole records, with sample size standardized at 735 individuals, we had estimated species richness of 22 and 12, respectively. Considering just adult records, with sample size standardized at 287 individuals, estimated species richness were 21 and 29, respectively. All results were very similar if we used just presence/absence records instead of abundances (27 and 29 for all records, 23 and 13 for tadpole records, 21 and 29 for adult records, respectively).

**Fig 3:**
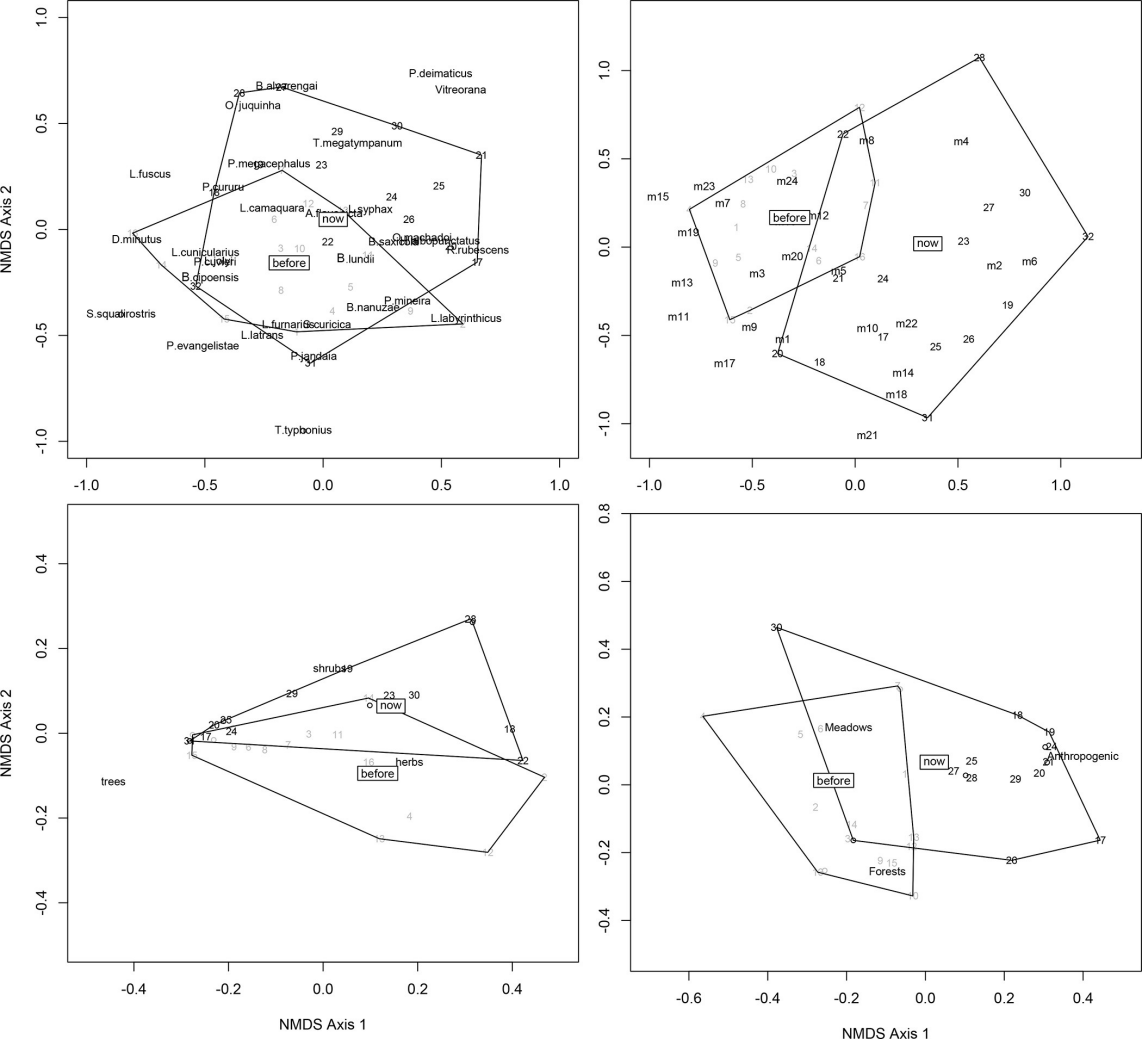
Distribution of species and environmental variables in space and time. Non-Metric Multidimensional Scaling (NMDS) showing (A) anuran species, (B) tadpole microhabitats, (C) presence of herbs, shrubs and tress in immediate riparian habitats, and (D) percent cover of land cover classes at 16 streams in two sampling periods (1998–1999 and 2015–2016) at Serra do Cipó, southeastern Brazil. Streams are numbered from 1 to 16 for the first sampling period and from 17 to 32 for the second sampling period.

All NMDSs reached solution (rmse = 0.001, max. resid. = 0.003 for species; rmse < 0.001; max. resid. < 0.001 for microhabitats, riparian vegetation strata, and land cover classes; Fig 3A–3D). Species composition differed between sampling periods (MS = 0.683, F = 2.391, df = 1, R^2^ = 0.060, p = 0.003). Differences in species composition were also significant among streams (MS = 0.433, F = 1.515, df = 15, R^2^ = 0.566, p = 0.004; Fig 2A). Only microhabitats 15–30 cm deep, with sandy bottom, vegetation, and no current (m14) explained very little of the variation in species composition (MS = 0.577, F = 1.576, df = 1, R^2^ = 0.048, p = 0.045). Microhabitat availability varied between sampling periods (MS = 1.805, F = 9.536, df = 1, R^2^ = 0.232, p = 0.001) but not among streams (MS = 0.208, F = 1.103, df = 15, R^2^ = 0.402, p = 0.269; Fig 3B).

Among the riparian vegetation strata, changes in the proportional cover of shrubs (MS = 0.538, F = 1.560, df = 1, R^2^ = 0.047, p = 0.044) and trees (MS = 0.924, F = 2.680, df = 1, R^2^ = 0.080, p = 0.001) explained some of the variation in species composition. Presence/absence of vegetation strata at stream margins varied among streams (MS = 0.033, F = 2.606, df = 15, R^2^ = 0.639, p = 0.018) and between sampling periods (MS = 0.091, F = 7.081, df = 1, R^2^ = 0.116, p = 0.005; Fig 3C).

Land cover classes did not explain a significant amount of variation in species composition (MS = 0.298, F = 0.825, df = 1, R^2^ = 0.026, p = 0.690 for natural forests, MS = 0.486, F = 1.342, df = 1, R^2^ = 0.042, p = 0.132 for montane meadows and MS = 0.543, F = 1.500, df = 1, R^2^ = 0.047, p = 0.071 for anthropogenic areas). Land cover classes did not vary among streams (MS = 0.049, F = 1.481, df = 15, R^2^ = 0.451, p = 0.124), but changed between sampling periods (MS = 0.399, F = 12.041, df = 1, R^2^ = 0.244, p = 0.001), showing increased human occupancy in 2015–2016 (Fig 3D).

**Fig 4:**
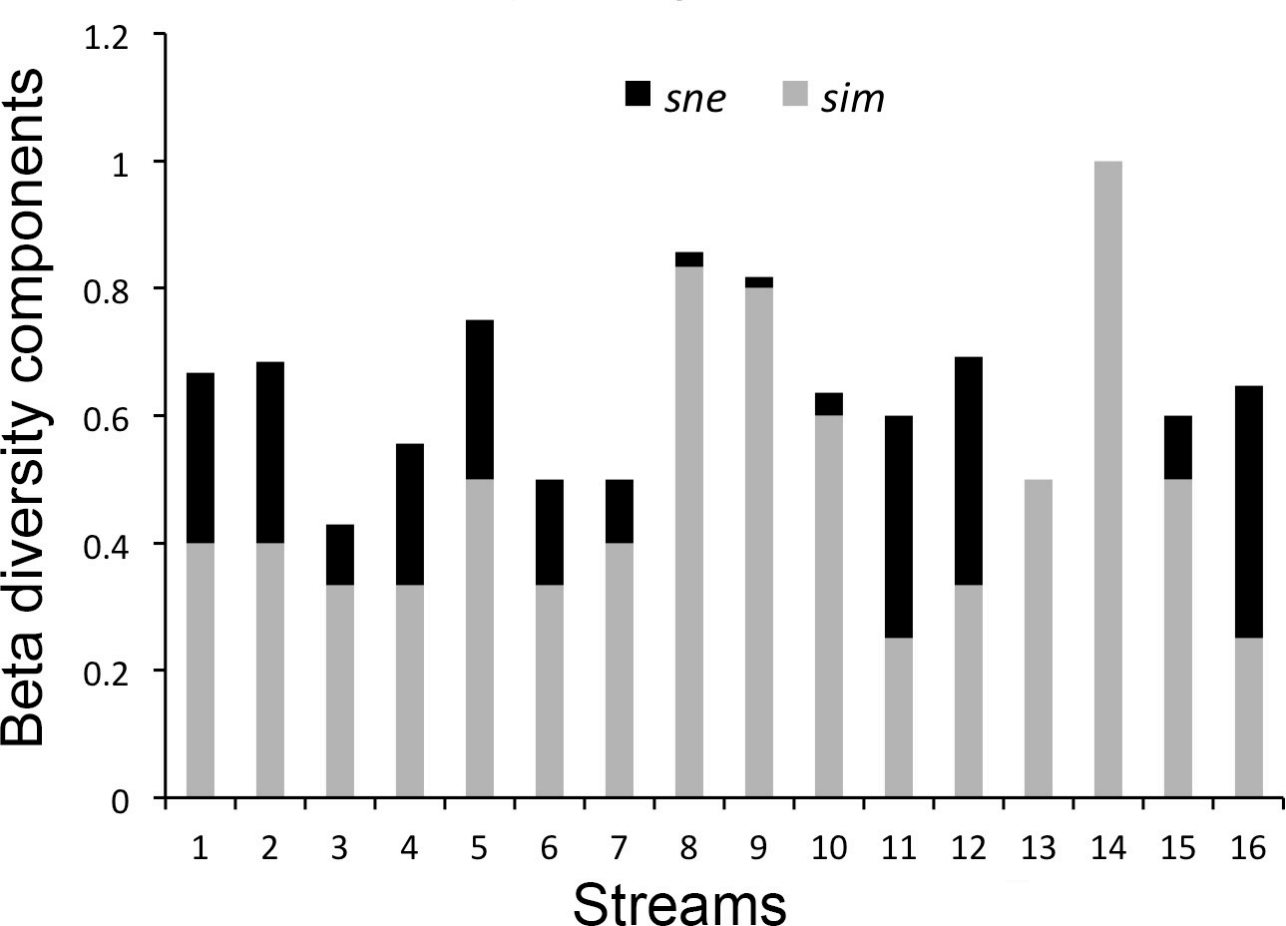
Anuran species turnover and nestedness. Contribution of turnover and nestedness components to variation in anuran species diversity between sampling periods in the 16 streams sampled at Serra do Cipó, southeastern Brazil.

We obtained 198 species/stream associations by summing every first record of a species presence at any stream in either sampling period. We used these data to assess variation in species diversity. From these species/stream associations, 96 occurred just during the 1998–1999 period, 62 just during the 2015–2016 period, and 40 were common to both periods (S3 Table). Variation in species diversity among streams for each time period included high turnover (1998–1999: β*sim* = 0.78; 2015–2016: *βsim* = 0.80) and low nestedness (1998–1999: *βsne* = 0.07; 2015–2016: β*sne* = 0.07). When we compared each stream between time periods the contribution of the components turnover and nestedness varied in importance (e.g., S8: β*sim* = 0.83, β*sne* = 0.02; S16: β*sim* = 0.25, β*sne* = 0.39; Fig 4).

**Fig 5:**
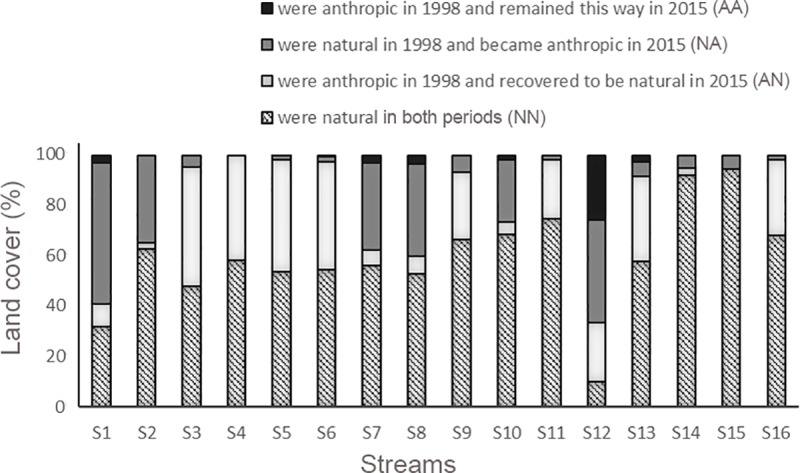
Changes in proportions of natural and anthropogenic land cover within 500-m buffers around 16 streams at Serra do Cipó, southeastern Brazil, from 1998 to 2015. The areas within the 500 m buffers around streams that were converted from natural to anthropogenic (NA) corresponded to 30–40% in S1, S2, S7, S8 and S12, 20% in S10 and less than 10% in the remaining streams (see also S2 Fig).

**Fig 6:**
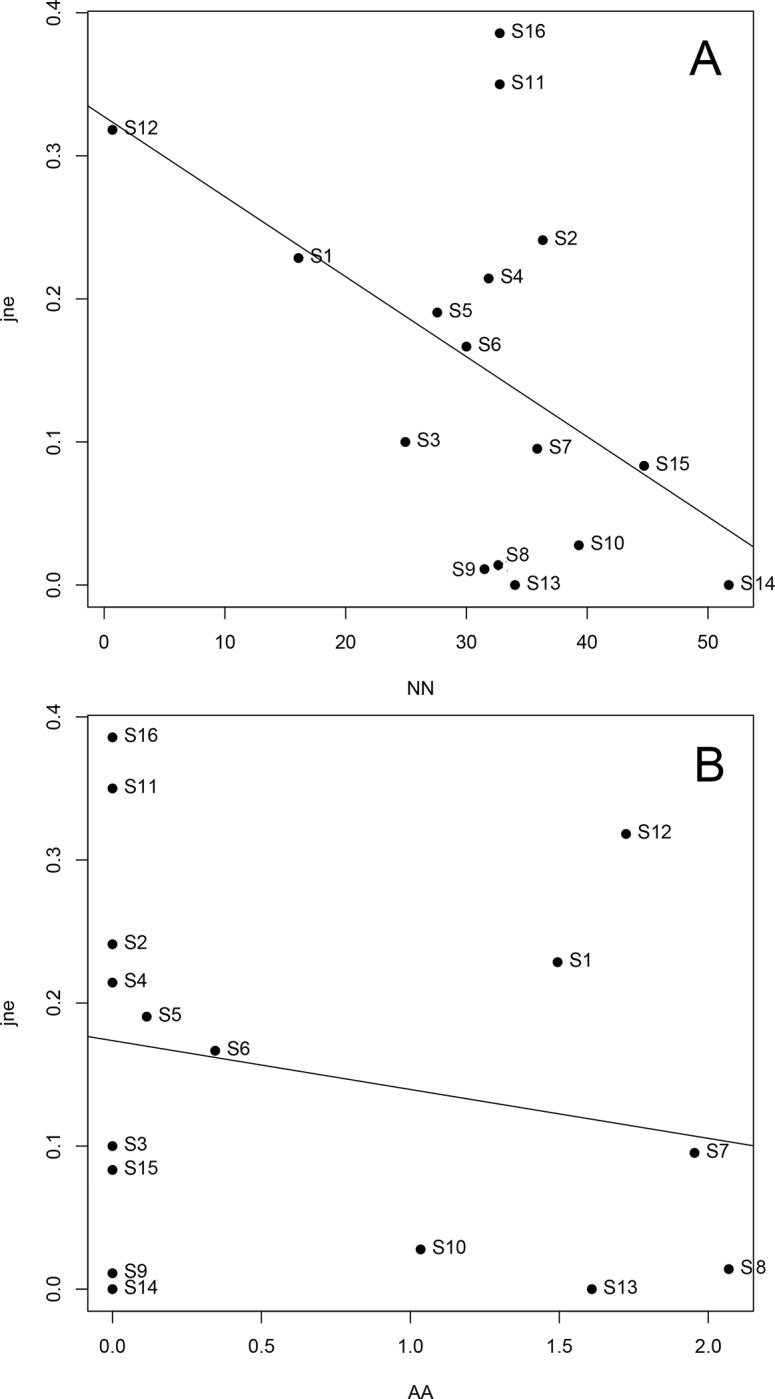
Relationships between nestedness and land cover. Relationships between nestedness (β*jne*) and proportion of (A) natural (NN) and (B) anthropogenic (AA) land cover that remained the same from 1998–1999 to 2015–2016 within 500-m buffers around 16 streams at Serra do Cipó, southeastern Brazil.

There was no spatial congruency among classes of land cover (S1 Fig). Areas that became anthropic (NA) did not explain species β-diversity or its components. Instead, streams with the largest proportions of natural or anthropogenic areas that were maintained from 1998 to 2015 (NN and AA, respectively) were those with the smallest values of nestedness (β*sne*; R^2^ = 0.454, r = 0.370, F = 5.4 on 2 and 13 df, p = 0.019; Figs 5 and 6). The other relationships between diversity components and classes of land cover change were not significant, although the relationship between β*sim* and NN was close to the adopted level of significance (β*sne*; R^2^ = 0.222, r = 0.166, F = 3.0 on 1 and 14 df, p = 0.065).

### Detectability and occupancy estimates

**Fig 7:**
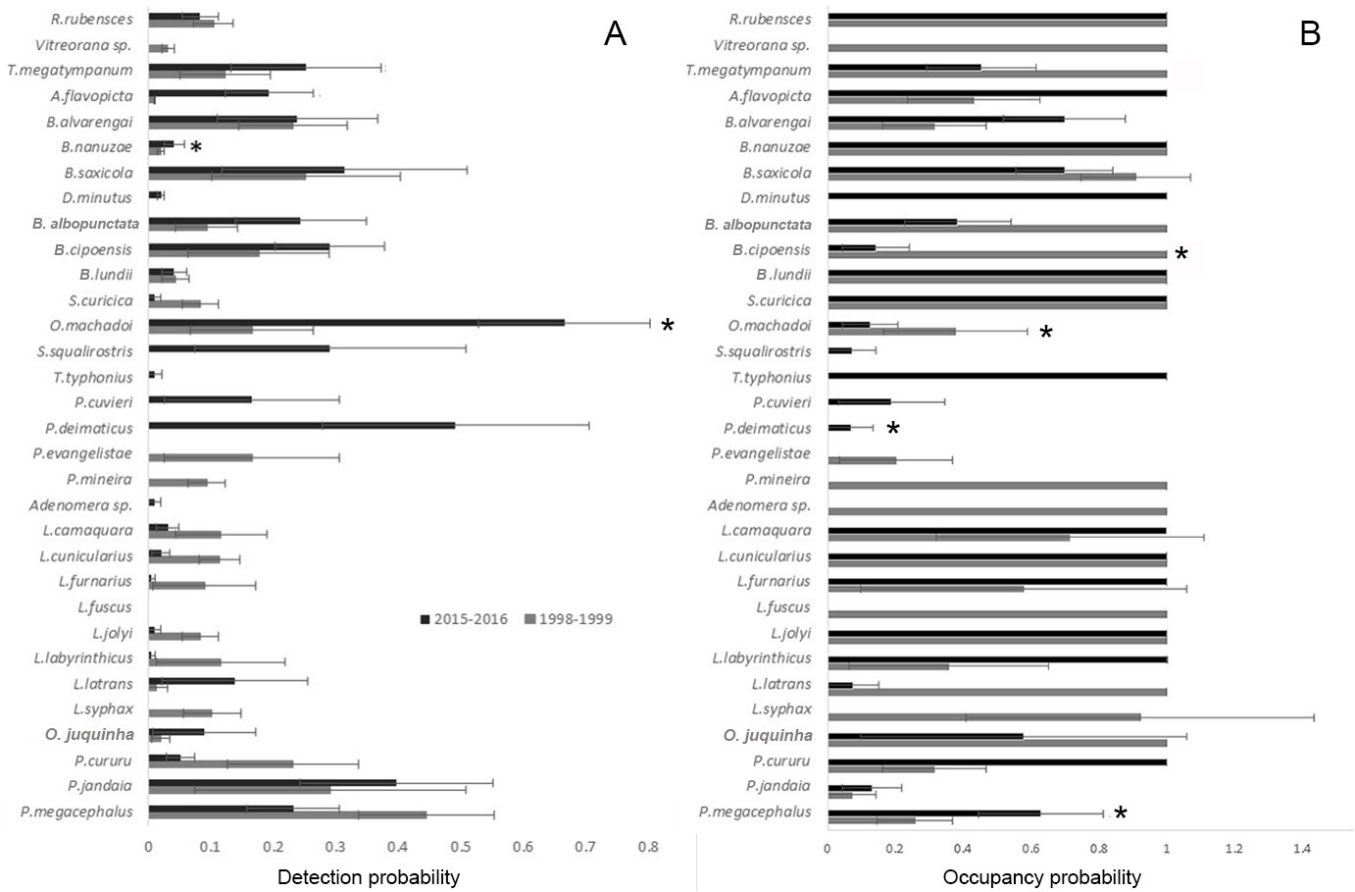
Anuran detection probabilities and stream occupancy estimates. Detection probabilities and stream occupancy estimates (with standard errors) of each anuran species in the sampling periods of 1998–1999 (dark grey) and 2015–2016 (light grey). * = significant differences at the level of p < 0.05.

We obtained 53 detectability estimates for all species in both sampling periods, of which only 23 were around or above 15% (considered adequate to obtain unbiased occupancy estimates according to [28,62]) and provided robust occupancy estimates (that is, the observed occupancy was included in the estimated *psi*(.) ± 1 SE(*psi*) interval [28]). Only five species had robust occupancy estimates in both sampling periods: *Bokermannohyla alvarengai*, *B*. *saxicola*, *Ololygon machadoi*, *Phasmahyla jandaia*, and *Pithecopus megacephalus* (Fig 7, S4 Table). Of these, only *O*. *machadoi* and *P*. *megacephalus* differed significantly between periods, the first with higher occupancy in 1998–1999 (t = 2.82, df = 10, p < 0.01) and the second in 2015–2016 (t = 4.22, df = 10, p < 0.001). Only *O*. *machadoi* differed significantly (p < 0.001) between periods in detection probability (Fig 5, S4 Table), with lower detection probability in 1998–1999 (although both observed and estimated occupancy values were higher in this period).

*Boana cipoensis* was recorded in 13 streams in 1998–1999 but only in three in 2015–2016. Although detectability values for this species were 18% and 30% for 1998–1999 and 2015–2016, respectively, the occupancy estimate was 100% (± 0.0) with a 95% confidence interval of 0–1 in 1998–1999. In this case such a result might be real and not an artifact of low detectability leading to imprecise occupancy estimates. Thus, the species could have been significantly more abundant in 1998–1999.

The number of streams with records decreased for *Boana lundii*, *Scinax curicica* and *Leptodactylus latrans* from 1998–1999 to 2015–2016. On the other hand, *Dendropsophus minutus*, *Scinax squalirostris*, *Trachycephalus typhonius*, *Physalaemus cuvieri*, *P*. *deimaticus*, *Adenomera* sp. and *Leptodactylus fuscus* were recorded just in 2015–2016 (considering the samples within the rainy season, S3 Table), and some species were recorded in more streams in 2015–2016 than in 1998–1999 (*Thoropa megatympanum*, *Ameerega flavopicta*, *Odontophrynus juquinha*, *Proceratophrys cururu*, and *Leptodactylus labyrinthicus*). Most of these species had low detectability (in one or both periods), hampering occupancy estimates/comparisons. For the ones with adequate detectability (*S*. *squalirostris*, *P*. *cuvieri*, and *P*. *deimaticus*), only *P*. *deimaticus* was expected to be detected with 95% certainty in the six visits accomplished (number of visits necessary for detection estimated as 4.4). Thus, this species was likely more widespread/abundant in 2015–2016.

Other remarkable changes in species composition/detection in the set of streams between sampling periods were the reductions in the number of streams with records of species of *Leptodactylus*, including *L*. *camaquara*, *L*. *cunicularius*, *L*. *furnarius*, *L*. *jolyi*, and *L*. *syphax* (S3 Table). However, all these *Leptodactylus* species had low detectabilities (S4 Table) and thus these results could be an artifact of imperfect sampling. *Vitreorana* sp., *Physalaemus evangelistae*, *Pseudopaludicola mineira* and *Leptodactylus syphax* were recorded in 1998–1999 but not in 2015–2016 (S3 Table). These species had low detectability, except for *P*. *evangelistae* (17%; S4 Table); however, 16–17 visits would be necessary to record this species presence in 1998–1999 with 95% certainty. Thus, *P*. *evangelistae* could have been present and remained undetected in 2015–2016 if detectability remained constant. The remaining species, *Rhinella rubescens*, *Bokermannohyla nanuzae*, and *Boana albopunctata*, had low and similar detectabilities in both sampling periods (S4 Table).

## Discussion

Estimated values of species richness in a set of 16 streams at Serra do Cipó, southeastern Brazil, seemed to increase from 1998–2000 to 2015–2016. However, the same sampling effort (based on sampling days) resulted in a larger number of individuals recorded in 2015–2016, which may be due to different weather conditions or chances of random encounters for species with low detectabilities. Among the seven species that were recorded just in 2015–2016, only *Dendropsophus minutus*, *Leptodactylus fuscus*, and *Physalaemus cuvieri* could be considered as widespread and resistant to impacted habitats [63]. Thus, increased human impact in the landscape could have favored these species, but this is not likely to be the only explanation for changes in estimated richness since species’ low detection probabilities are also expected to play a role in these results. When we standardized sampling effort based on number of recorded individuals, species richness seemed equivalent in the two time periods. The records of tadpole and adult individuals were complementary for species inventory as also shown by [54], with the record of tadpoles aiding further to species inventory in 1998–1999. However, numbers of individuals detected in either life stage did not influence our results, considering that estimates based on both abundance and presence/absence data yielded very similar results.

Species composition, on the other hand, changed considerably between the two time periods (see S3 Table). There was no apparent relationship between natural changes in microhabitat availability and changes in species composition at individual streams. However, changes in cover of shrubs and trees in the immediate riparian habitat influenced species composition. At the landscape level, changes in types of land cover (from natural to anthropogenic and in the other direction) seemed to cause species extinctions and colonizations, with colonizations likely being hampered by increased habitat destruction in the matrix (see [64]), causing nestedness to increase. The proportion of variation in species composition explained by habitat changes was small, indicating that migration/extinction dynamics (whether random or responding to any environmental cue) may explain an important part of local species composition at streams. All these results, though, must be interpreted with caution due to the low detectabilities estimated for most species.

At the finest spatial scale considered, just one microhabitat type explained a small amount of variation in species composition. Availability of potential tadpole food items has been shown to vary among microhabitats in streams (based on another study conducted at streams at the same site [65]) and food items provide different nutrients for tadpoles, influencing their growth and metamorphosis (e.g. [66–67]). Microhabitats can also vary in their effectiveness in providing protection for tadpoles from predation (e.g. [68]). Thus, structural variation at the microscale is likely to influence tadpole metamorphosis and/or survivorship, ultimately affecting species presence at a given locality. However, reduced tadpole abundance could affect detectability [69], and also contribute to the observed variation in species composition. Our detailed classification of tadpole microhabitats may have precluded the association of a significant amount of changes in species composition to a single microhabitat type. Besides, microhabitats used by tadpoles greatly overlap in availability of food types [65]. Thus, only a broad alteration of stream microstructure might be expected to reduce the set of available microhabitats in a way to cause noticeable effects on anuran species composition via decreased resource availability.

On the other hand, the amount of shrubs and trees in the immediate riparian habitat varied among streams and between time periods, explaining a significant amount of variation in species composition. We could not observe any evidence of anthropic origin for such variation with the exception of S1, where records of shrubs and trees increased at the vicinities of a hotel from 1998–2000 to 2015–2016, and the number of anuran species decreased from 14 to six. Anuran population trajectories in urban and rural areas can be strongly dependent on the amount of vegetation in the aquatic habitat and its surroundings [70]. Removal of arboreal vegetation in urban and other modified areas led to the disappearance of some amphibian species [11] and caused reduced species richness [71]. Many montane meadow frog species, however, may prefer open riparian habitats for breeding at the study site [42]. Additionally, stream colonization events by species adapted to open areas may be likely and frequent, although little is known about frog migration in montane meadow habitats [35–36].

Although land cover classes did not explain changes in species composition at the broadest scale considered, anthropogenic areas were clearly more widespread in 2015–2016 (see Fig 3D). If we consider just the species that had adequate detectability and robust occupancy estimates, we detected significant changes in occupancy between periods for four out of seven species. Of these same seven species, three were recorded in S1 during 1998–1999 and disappeared in 2015–2016 (*Boana cipoensis*, *Phitecopus megacephalus*, and *Phasmahyla jandaia*; S2 Table). It is possible that imperfect sampling due to the low detectability of most species precluded the association of a more widespread effect of anthropogenic changes on the composition of anuran assemblages.

Species turnover was the main component of beta diversity among streams and at particular streams between sampling periods. Stream variables such as riparian vegetation, land cover, shelter/microhabitat and breeding habitat availability are important in determining anuran species composition ([34, 72–73]; present study). All these variables represent gradients, along which species have different preferences/adaptations (see [42] for the studied species). Thus, high turnover compared to nestedness would be expected among the studied streams, since they varied both spatially and temporally in riparian vegetation structure and temporally in tadpole microhabitat availability and representativeness of land cover classes.

When comparing specific streams between time periods, some species are apparently being replaced by others instead of just being lost, considering that even in the streams with the highest values of nestedness, this component is still considerably lower than species turnover, except for S11 (β*sim* = 0.25, β*sne =* 0.35) and S16 (β*sim* = 0.25, β*sne* = 0.39). Other streams in which nestedness was representative were S1 (β*sne* = 0.26), S2 (β*sne* = 0.28), and S12 (β*sne* = 0.35), which also had the greatest anthropization in their surroundings from 1998–1999 to 2015–2016. Anthropization may be one cause of increased nestedness, although other reasons may be natural or random. Streams S11 and S16 had high nestedness and little anthropogenic land cover, whereas S7 and S8 suffered considerable anthropization but had low nestedness (see Figs 4 and 5). Greater areas with natural cover in both time intervals (NN) combined with unchanged areas of anthropogenic cover (AA) were related to lower values of nestedness. This probably means that nestedness may increase with increased levels of environmental change (represented by the other complementary categories, NA and AN). Changes in any direction along a gradient are likely to induce negatively affected species to abandon a site or may favor colonization of species positively affected by such changes. Amphibian species composition was shown to vary throughout a gradient of anthropogenic impacts [74], although species richness did not reflect such changes. This result was interpreted as the loss of species sensitive to the disturbances concomitantly with the dispersion of species tolerant to them [74]. However, there should be a point where human induced changes may decrease local diversity beyond natural levels by combined local extinctions and less likely colonizations through a disturbed matrix (e.g. [3, 75]). Thus, in landscapes where most species are sensitive to ongoing disturbances, species losses are likely to overcome arrivals of tolerant species.

Among the species with robust occupancy estimates, *Ololygon machadoi* and *Boana cipoensis* showed a reduction in occupancy in 2015–2016 compared to 1998–1999, whereas *Pithecopus megacephalus* and *Physalaemus deimaticus* increased their occupancy in the same interval. Such alterations were not associated to any particular class of land cover change and could represent natural random fluctuations. There is no evidence for decline of any species so far between the studied time intervals, and we call attention to an important issue in monitoring studies of montane meadow stream frog assemblages: most species have low abundances and detectability, requiring a great number of sampling visits for detection (see S4 Table). Even decreases in occupancy, such as observed here for *O*. *machadoi* and *B*. *cipoensis*, as well as those observed by [14] for *A*. *flavopicta* and *Crossodactylus bokermanni* from 1971–1974 to 1996–2002, could be a result of natural population fluctuations in a dynamic landscape. Population fluctuations are not necessarily associated with extinction probability [76], but can result from the interaction of a species with its habitat and the connectivity of the landscape [77].

On the other hand, species rarity/low detectability may hamper the detection of real declines if they happen to occur. Species rarity is likely to reduce detectability [69] and produce high occupancy rates due to lack of precision of the estimates (e.g., *Leptodactylus camaquara*, *L*. *cunicularius*, *L*. *furnarius*, *L*. *jolyi*, and *L*. *syphax* in this study). In such cases, the hypothesis that species suffered population declines in the time interval considered cannot be discarded, although it cannot be corroborated by the data either. Furthermore, even if species do not show detectable changes in occupancy, genetic diversity of local populations is likely to be lost if anthropogenic alterations in the landscape hamper frog migration [35].

We showed that spatial and temporal changes in environmental features from the microhabitat to the landscape level can all explain some proportion of variation in species composition and diversity. Human induced changes are just one more variable likely to affect frog species composition in dynamic montane meadow stream assemblages. This fact, combined with low detectability of most species even under intensive thorough field inventories, makes it difficult to pinpoint real cases of amphibian population declines. We point to the need to develop new, stronger monitoring protocols and analyses, as well as to test the efficacy of new techniques, such as environmental DNA (see [69]), as potentially more efficient tools regarding species detectability in diverse tropical amphibian communities in comparison to traditional field inventories.

## Supporting information

S1 Table Information on frog specimens in adult and tadpole stages collected during field inventory at Serra do Cipó, Southeastern Brazil. Museum numbers and corresponding species of all tadpoles and frogs collected during the study are presented.
(XLSX)

S2 Table Description of studied stream sections regarding immediate riparian vegetation, microhabitat availability for tadpoles, and percent land cover represented by anthropogenic habitats, native forests, and montane meadows. See methods for details on data acquisition.
(XLSX)

S3 Table Number of adult frogs and tadpoles of 32 species recorded in 16 streams at Serra do Cipó, southeastern Brazil, during standardized sampling periods (34 sampling days) in 1998–1999 and 2015–2016. Eggs and froglets were also recorded during the study, but did not represent new records for any stream, thus are not represented.
(XLSX)

S4 Table Detectability (p) and occupancy (psi) results for 32 anuran species at 16 streams at Serra do Cipó, southeastern Brazil. Real detection during six samplings per stream within the rainy season (naive), occupancy and detectability estimates obtained with the software PRESENCE and estimated number of visits necessary for 95% detection probability of a species once present at a given site are presented. Detectability values high enough to provide robust estimates of occupancy are in boldface. * indicates occupancy values that differed significantly between sampling periods (1998–1999 and 2015–2016), only considered for species with detection probabilities of around 15% or higher in both periods (see text for details).
(XLSX)

S1 Fig Test of spatial autocorrelation for three types of land cover: anthropogenic (A), montane meadow (B), and natural forests (C).
(TIF)

S2 Fig Image classification for 500-m buffers around 16 sampled stream sections (S1 –S16) for the years of 1998 and 2015 showing anthropogenic areas (dark blue), montane meadows (light blue), natural forests (green), clouds (red), and shadows (beige). Turnover values between time periods are represented at the left of each stream.
(TIF)
