# Supplementary figures and images for: Dynamic and diverse amphibian assemblages: Can we differentiate natural processes from human induced changes?
Source: PLoS One. 2019 Mar 26;14(3):e0214316. doi: 10.1371/journal.pone.0214316 (PMC6435182; doi:10.1371/journal.pone.0214316)

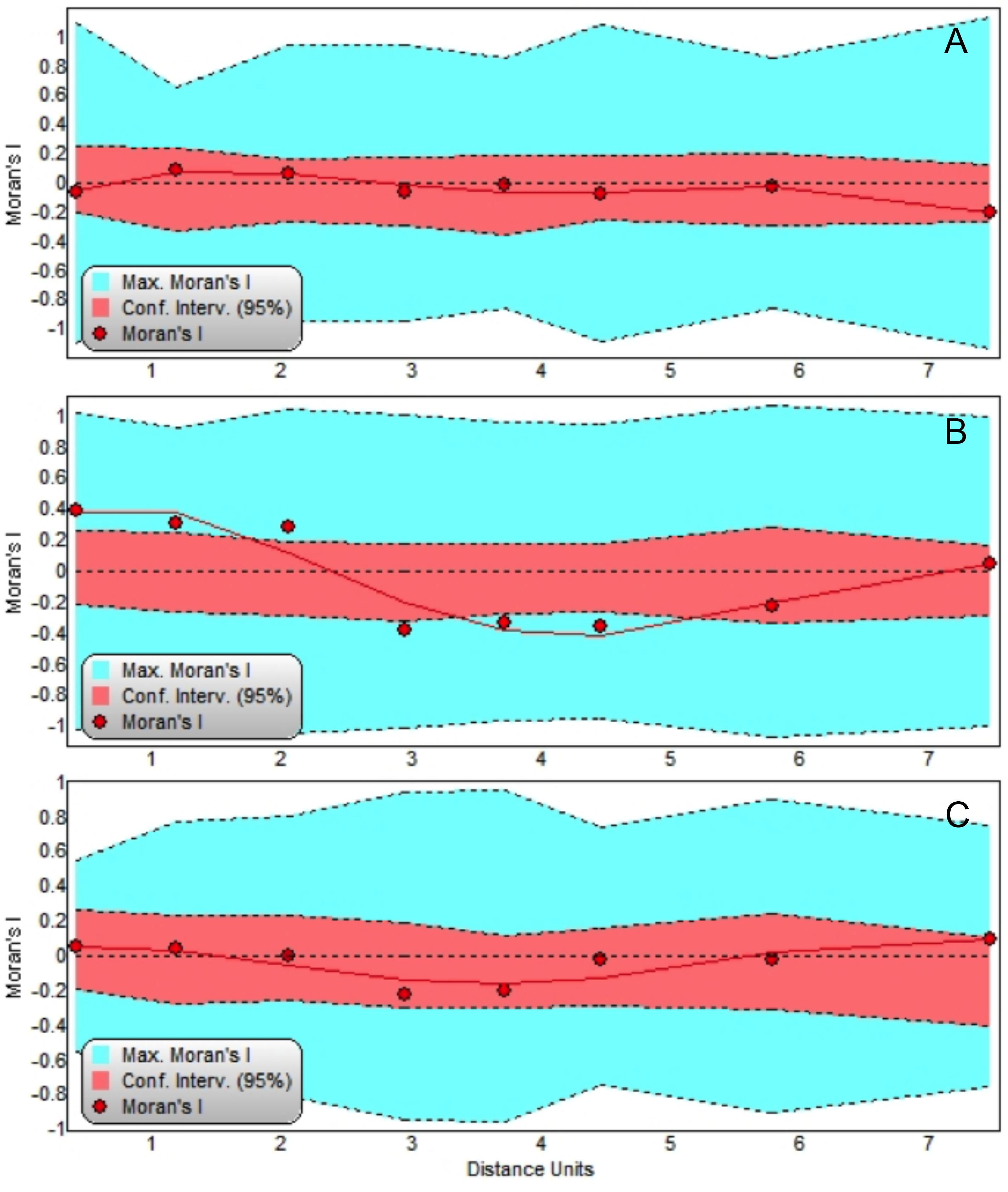

Supplement: S1 Fig — Test of spatial autocorrelation for three types of land cover: anthropogenic (A), montane meadow (B), and natural forests (C). (TIF) [file pone.0214316.s005.tif]

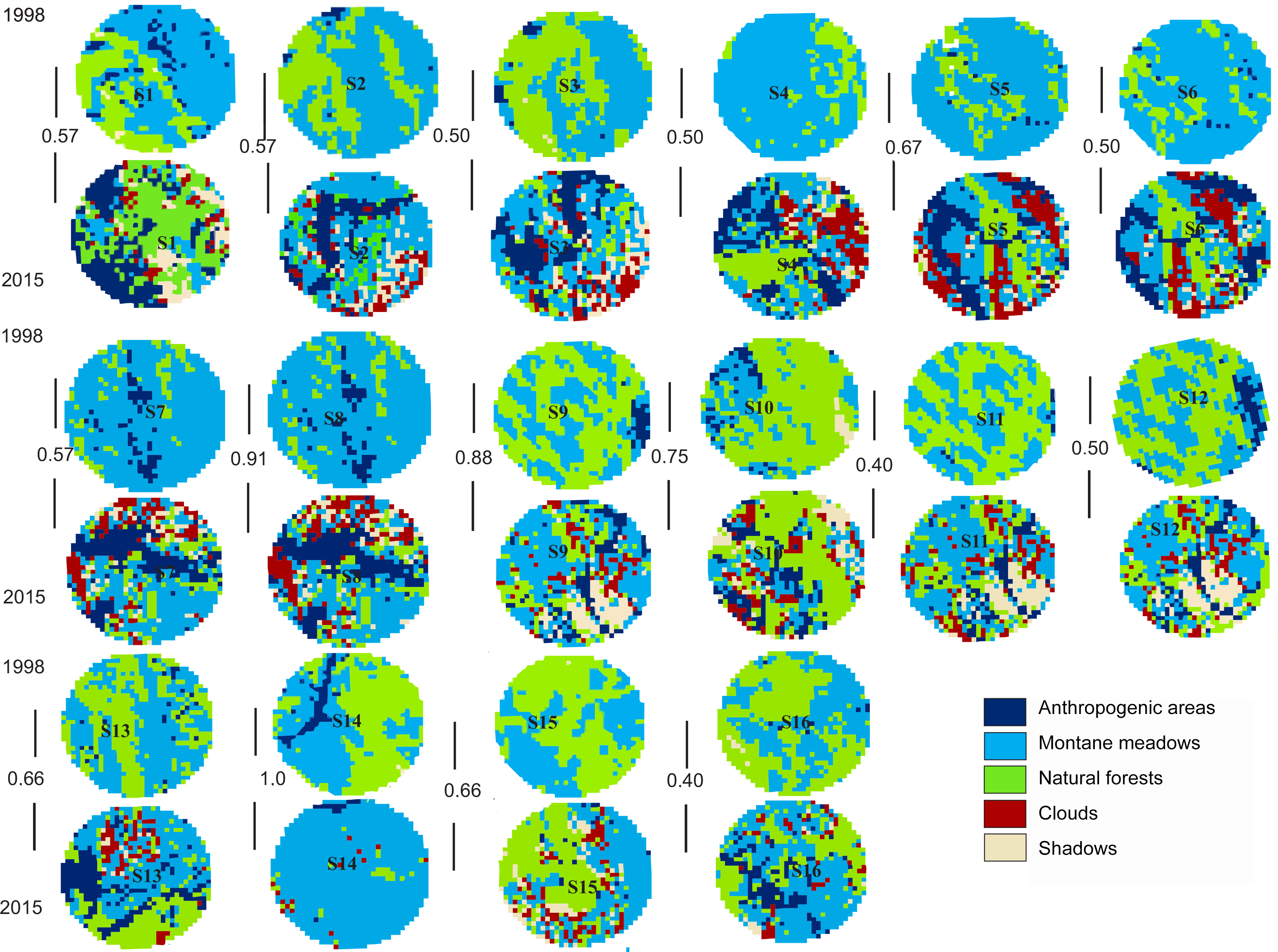

Supplement: S2 Fig — Turnover values between time periods are represented at the left of each stream. (TIF) [file pone.0214316.s006.tif]
